# Complete genome sequence and comparative genome analysis of *Klebsiella oxytoca* HKOPL1 isolated from giant panda feces

**DOI:** 10.1186/1756-0500-7-827

**Published:** 2014-11-23

**Authors:** Jingwei Jiang, Hein Min Tun, Nathalie France Mauroo, Angel Po Yee Ma, San Yuen Chan, Frederick C Leung

**Affiliations:** Bioinformatics Center, Nanjing Agricultural University, Nanjing, China; School of Biological Sciences, The University of Hong Kong, Hong Kong SAR, China; Department of Pathology, The University of Hong Kong, Hong Kong SAR, China; Clinical Laboratory, Veterinary Center, Ocean Park Corporation, Hong Kong SAR, China; Gut Microbiome and Large Animal Biosecurity Laboratories, Department of Animal Science, University of Manitoba, Winnipeg, MB Canada

**Keywords:** *Klebsiella oxytoca*, Giant panda, Complete genome sequence, Gut microbiota, Cellulose degradation, Biofuel

## Abstract

**Background:**

The giant panda (*Ailuropoda melanoleuca)* is an endangered species well-known for ingesting bamboo as a major part of their diet despite the fact that it belongs to order *Carnivora*. However, the giant panda’s draft genome shows no direct evidence of enzymatic genes responsible for cellulose digestion. To explore this phenomenon, we study the giant panda’s gut microbiota using genomic approaches in order to better understand their physiological processes as well as any potential microbial cellulose digestion processes.

**Results:**

A complete genome of isolated *Klebsiella oxytoca* HKOPL1 of 5.9 Mb has been successfully sequenced, closed and comprehensively annotated against various databases. Genome comparisons within the *Klebsiella* genus and *K. oxytoca* species have also been performed. A total of 5,772 genes were predicted, and among them, 211 potential virulence genes, 35 pathogenicity island-like regions, 1,615 potential horizontal transferring genes, 23 potential antibiotics resistant genes, a potential prophage integrated region, 8 genes in 2,3-Butanediol production pathway and 3 genes in the cellulose degradation pathway could be identified and discussed based on the comparative genomic studies between the complete genome sequence of *K. oxytoca* HKOPL1 and other *Klebsiella* strains. A functional study shows that *K. oxytoca* HKOPL1 can degrade cellulose within 72 hours. Phylogenomic studies indicate that *K. oxytoca* HKOPL1 is clustered with *K. oxytoca* strains 1686 and E718.

**Conclusions:**

*K. oxytoca* HKOPL1 is a gram-negative bacterium able to degrade cellulose. We report here the first complete genome sequence of *K. oxytoca* isolated from giant panda feces. These studies have provided further insight into the role of gut microbiota in giant panda digestive physiology. In addition, *K. oxytoca* HKOPL1 has the potential for biofuel application in terms of cellulose degradation and potential for the production of 2,3-Butanediol (an important industrial raw material).

**Electronic supplementary material:**

The online version of this article (doi:10.1186/1756-0500-7-827) contains supplementary material, which is available to authorized users.

## Background

The giant panda (*Ailuropoda melanoleuca)* is an endangered species with a population of less than 2,500 in the wild and approximately 200 in zoological institutions across the world. Their natural habitats are located in the western provinces of China [1]. The giant panda is a carnivore well-known for ingesting bamboo as part of their major diet. The giant panda’s draft genome disclosed no direct evidence of enzymatic genes responsible for cellulose digestion [2], which leads us to believe that its digestion of bamboo may rely on its gut microbiota [2]. To understand microbial cellulose digestion and how it affects the physiological processes of giant pandas, it is very important to study their gut microbiota. Although a previous metagenomic study reported evidence of cellulose metabolism in the gut of giant pandas [3], genomic studies of their gut microbiota are still lacking. Our previous 16s rDNA amplicon study showed that *Proteobacteria* and *Firmicutes* are two major bacterial components in 4 giant pandas [4]. It also revealed that the *Klebsiella* genus is one of 40 core OTUs shared between them [4].

There are approximately 2,600 complete bacteria genome sequences available in the NCBI GenBank (October 2013). For the *Klebsiella* genus, there are only nine complete genome sequences, including six *Klebsiella pneumoniae,* a *Klebsiella variicola*, and two *Klebsiella oxytoca. Klebsiella pneumoniae subsp. pneumoniae* HS11286 and *Klebsiella pneumoniae* NTUH-K2044 are two important pathogen causing various opportunistic infections [5, 6], *Klebsiella pneumoniae Strain* KCTC 2242 is a 2,3-Butanediol (2,3-BD) producing, industrially important bacterium [7], *Klebsiella pneumoniae* 342 is a nitrogen-fixing bacterium [8], *Klebsiella variicola* At-22 is a nitrogen-fixing bacterium [9], and *Klebsiella oxytoca*, ubiquitous in nature, can be found in various environments [10]. In previous reports, *K. oxytoca* strains were also characterized by their nitrogen fixation [11, 12] and cellulose hydrolyzation [12] abilities.

In this study, we first isolated the resident bacterium *K. oxytoca* HKOPL1 from the gut of a giant panda residing in a zoological institution of Hong Kong. To obtain the complete genome sequence of this bacterium, we applied the bacterial genome sequencing strategy proposed in our previous simulation study. The resulting complete genome sequence was then used to perform bioinformatic analysis and functional investigations.

## Results and discussion

### Sequencing and assembly of the *Klebsiella oxytoca*HKOPL1 complete genome

For the 454 shotgun runs, the first sequencing run generated 110,684 reads (~50 Mb of sequence information) and the other generated 82,726 reads (~35 Mb of sequence information). Both of these shotgun runs generated ~10× shotgun reads and the average read length is about 400 bp. For the 454 paired-end runs, the first sequencing run generated 116,008 reads (~38 Mb of sequence information) and the other generated 121,685 reads (~46 Mb of sequence information). Both of the two paired end runs generated ~10× paired end reads and the average read length is about 330 bp. The raw shotgun reads and paired-end reads were assembled into 123 contigs and 3 scaffolds. The N50 contig size is 145,039 bp and the largest scaffold contains 68 contigs contributing to a size of 5,911,648 bp, which shows that our raw assembly is highly contiguous. Over 99% of the total reads were assembled, resulting in coverage of around 23× for *Klebsiella oxytoca* HKOPL1. After PCR gap filling and Sanger sequencing of the PCR product, the complete genome of *Klebsiella oxytoca* HKOPL1 was achieved.

We hereby report that the genome of *Klebsiella oxytoca* HKOPL1 is a circular genome of 5.9 Mb with 55.92% GC content (Table 1). This data is now available in the NCBI GenBank (accession: CP004887, BioProject ID: PRJNA194061).

**Table 1:** **General features of the complete genome of** ***K. oxytoca*** **HKOPL1**

Feature	Chromosome
Length (bp)	5914407
GC content (%)	55.92
# of predicted genes	5772
# of annotated genes	5528
# of tRNA genes	35
# of rRNA genes	62

### Genome annotation of *Klebsiella oxytoca*HKOPL1

**Figure 1:**
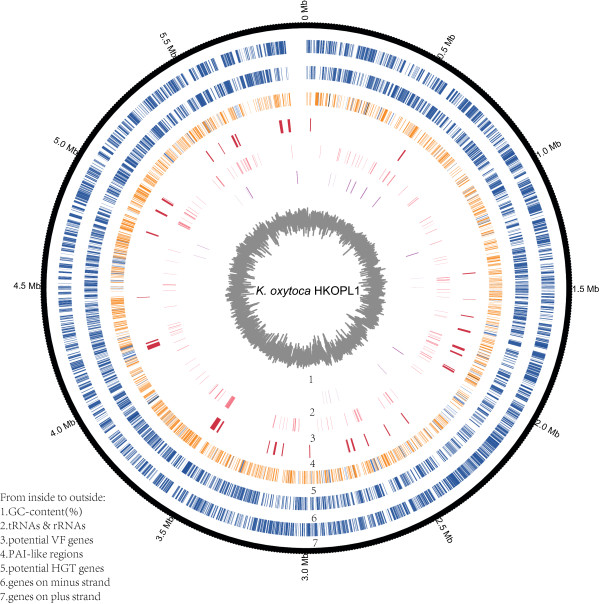
**Genome features of** ***K. oxytoca*** **HKOPL1** ***.*** From inside to outside, 1: GC-content (%); 2: tRNA & rRNA elements (purple); 3: Potential virulent factors (pink); 4: PAI-like regions (red); 5: Potential HGT genes (orange: plasmid genes, blue: prophage genes and black: prophage associated genes); 6: Genes on minus strand and 7: Genes on plus strand.

A total of 5,772 CDS sequences were predicted from the complete *K. oxytoca* HKOPL1 genome. About 95.8% (5,528 genes) of the total predicted genes were annotated against the NCBI non-redundant database (Table 1). Thirty-five tRNAs and sixty-two rRNAs were identified in the chromosome sequence. Most RNA elements were clustered to form 6 large RNA islands (Figure 1).

### Phylogenomic analysis of *Klebsiella oxytoca*HKOPL1

**Figure 2:**
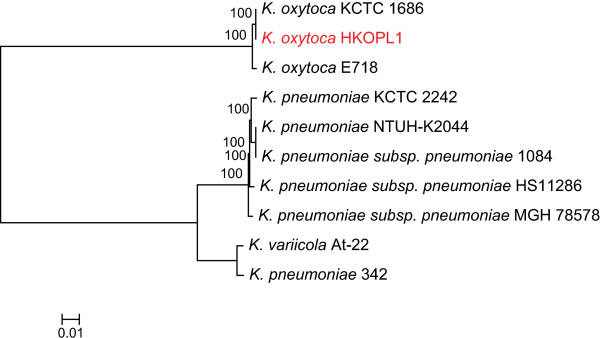
**BMCMC Phylogenomic tree (1391 concatenated core genes) of 10** ***Klebsiella*** **strains (Posterior probabilities were presented on the nodes).**

After comparative genome analysis, 1,391 genes were identified as core genes from 10 complete *Klebsiella* strains. Subsequently, a BMCMC (Bayesian Markov Chain Monte Carlo) phylogenomic tree was constructed based on these identified core genes (Figure 2). Our phylogenomic analysis highlighted that the *Klebsiella oxytoca* HKOPL1 strain is clustered with *K. oxytoca* strains 1686 and E718, and it is phylogenetically distant from the *K. pneumoniae* strains (Figure 2). It is also phylogenetically closer to *K. oxytoca KCTC* 1686 than *K. oxytoca* E718 (Figure 2).

**Figure 3:**
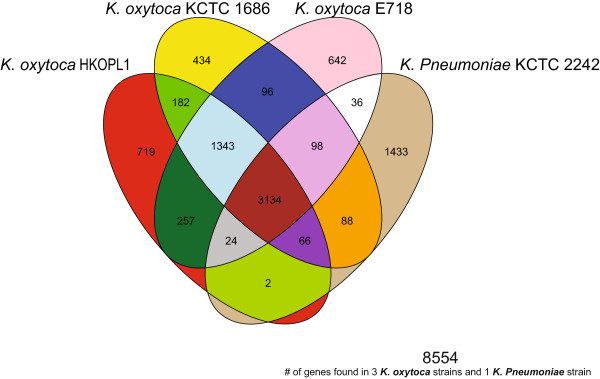
**Venn diagram based on the number of common genes found in the 3** ***K. oxytoca*** **strains and 1** ***K. pneumonia*** **strain.**

*K. oxytoca* has a different classification from *K. pneumoniae* because it can grow on melezitose, but not on 3-hydroxybutyrate [10]; *K. oxytoca* is also phylogenetically distant from *K. pneumonia* based on 1,391 core genes, indicating that *K. oxytoca* may have many other unique features*.* After comparative analysis between the three *K. oxytoca* strains and one representative *K. pneumoniae* strain *(K. pneumoniae* KCTC 2242*)*, we put together a venn diagram showing 3,134 common genes shared among the four strains (36.7% out of 8,554 genes found in all four *Klebsiella* strains, see Figure 3). *K. pneumoniae* KCTC 2242 has more strain-specific genes and shares less common genes (<100) with the other *K. oxytoca* strains, while the three *K. oxytoca* strains share 1,343 common genes absent in the genome of *K. pneumoniae* KCTC 2242 (Figure 3).

The numbers of predicted genes in the chromosomes of all *Klebsiella* strains range from 4,923 to 5,772 (see Additional file 1); the percentage of paired orthologous genes in all *Klebsiella* strains shared with *K. oxytoca* HKOPL1 ranges from 37.85% to 86.22% (see Additional file 1). Additional file 1 also indicates that the percentage of paired orthologous genes in *K. oxytoca* strains 1686 and E718 (>80%) is much higher than that in both *K. pneumonia* and *K. variicola* strains (~40%). Additional file 2 suggests that all paired orthologous genes in *Klebsiella* strains are shared with *K. oxytoca* HKOPL1*,* and that their PAI-like regions and prophage-like regions also share similar positioning.

Both phylogenomic analysis and the percentage of common genes in all *Klebsiella* strains show that *K. oxytoca* HKOPL1 is closer to *K. oxytoca* strains 1686 and E718*,* rather than *K. pneumoniae* and *K. variicola* strains.

### COG annotation and comparative analysis between *Klebsiella oxytoca*HKOPL1 and *K. oxytoca*strains 1686 and E718

**Figure 4:**
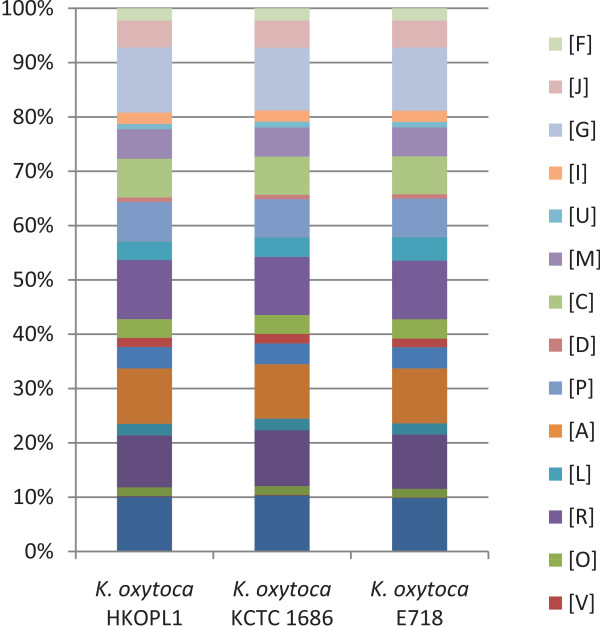
**Comparative single letter COG analysis for** ***K. oxytoca*** **HKOPL1 and other closely related strains.** Function of each single letter COG: [F] Nucleotide transport and metabolism; [J] Translation, ribosomal structure and biogenesis; [G] Carbohydrate transport and metabolism; [I] Lipid transport and metabolism; [U] Intracellular trafficking, secretion, and vesicular transport; [M] Cell wall/membrane/envelope biogenesis; [C] Energy production and conversion; [D] Cell cycle control, cell division, chromosome partitioning; [P] Inorganic ion transport and metabolism; [A] RNA processing and modification; [L] Replication, recombination and repair; [R] General function prediction only; [O] Posttranslational modification, protein turnover, chaperones; [V] Defense mechanisms.

A total of 4,337 (~75.1%) out of 5,772 predicted genes of *K. oxytoca* HKOPL1 were identified in the NCBI COG database. Comparative study of single letter COG analysis for the three closely related *K. oxytoca* strains is shown in Figure 4, and the result for *K. oxytoca* HKOPL1 shows that the 9 COG classes ([G] (10.15%), [K] (8.58%), [S] (8.12%), [E] (8.65%), [R] (9.2%), [P] (6.13%), [C] (6.04%), [M] (4.61%) and [J] (4.2%)) have over 4% of the total annotated COG genes that comprise over half of the total annotated COG genes. The corresponding functions of these 9 COG classes are: carbohydrate transport and metabolism [G], transcription [K], function unknown [S], amino acid transport and metabolism [E], general function prediction only [R], inorganic ion transport and metabolism [P], energy production and conversion [C], cell wall/membrane/envelope biogenesis [M] and translation, ribosomal structure and biogenesis [J]. After the comparative COG study, the percentages of all COG classes were found to be similar within the three *K. oxytoca* strains (Figure 4).

### Virulence gene/pathogenicity island-like region annotation and comparative virulence gene analysis of *K. oxytoca*HKOPL1 with other *Klebsiella*strains

Only 211 (~3.7%) out of 5,772 predicted genes of *K. oxytoca* HKOPL1 were identified in the VFDB database. Comparative study of virulence genes between *K. oxytoca* HKOPL1 and other *Klebsiella* strains shows that all *Klebsiella* strains have 3.5%-4.07% of virulence genes in their genomic sequences (Table 2); the location of all potential virulence genes in the *K. oxytoca* HKOPL1 genome is shown in Figure 1. Thirty-five Pathogenicity island-like regions were identified in the chromosome and their features are shown in Additional file 3.

**Table 2:** **Comparative study of the potential virulence genes for** ***K. oxytoca*** **HKOPL1 and other** ***Klebsiella*** **strains**

Strain name	Accession	# of genes	# of virulence genes	% of virulence genes
*K. oxytoca* HKOPL1	CP004887	5772	216	3.74
*K. oxytoca* KCTC 1686	NC_016612	5488	209	3.81
*K. oxytoca* E718	NC_018106	5701	204	3.58
*K. pneumoniae subsp. pneumoniae* MGH 78578	NC_009648	4776	181	3.79
*K. pneumoniae* 342	NC_011283	5425	190	3.50
*K. pneumoniae* NTUH-K2044	NC_012731	4992	202	4.05
*K. variicola* At-22	NC_013850	5057	185	3.66
*K. pneumoniae subsp. pneumoniae* HS11286	NC_016845	5316	191	3.59
*K. pneumoniae* KCTC 2242	NC_017540	4923	185	3.76
*K. pneumoniae subsp. pneumoniae* 108	NC_018522	4962	202	4.07

*K. oxytoca* was reported as a causative organism of antibiotic-associated Hemorrhagic Colitis in human patients [13]. However, their pathogenicity in giant pandas is still unknown. In the *K. oxytoca* HKOPL1 genome, 35 pathogenicity island-like regions were identified, of which 14, PAI-2, PAI-5, PAI-7, PAI-8, PAI-9, PAI-11, PAI-14, PAI-18, PAI-20, PAI-21, PAI-22, PAI-24, PAI-27 and PAI-33, make up over 50% of PAI-virulence genes out of the total number of ORFs (see Figure 1 and Additional file 3).

A comparative CDS study (see Additional file 2) indicates that PAI-5 exists in all *Klebsiella* strains, while PAI-2, PAI-8, PAI-9, PAI-11, PAI-14, PAI-18, PAI-20, PAI-21, PAI-24 and PAI-33 only exist in *K. oxytoca* strains. PAI-7, PAI-22 and PAI-27 exist in all *K. oxytoca* strains, and some of these genes are partially shared among other *Klebsiella* strains. Although virulence genes and pathogenicity island-like regions were observed in the *K. oxytoca* HKOPL1 genome, over-interpretation of its pathogenicity should be avoided (especially as the strain was isolated from a healthy giant panda) and further targeted fragment knockout experiments are required for confirmation.

### Drug resistance genes and potential antibiotic resistance

Only 23 (~0.4%) out of 5,772 predicted genes of *K. oxytoca* HKOPL1 were identified in the ARDB database and were matched to their corresponding resistant antibiotics. A comparative study of *K. oxytoca* HKOPL1 and two other closely related *K. oxytoca* strains shows that all three strains potentially have antibiotic resistance against gentamicin_b, acriflavin, chloramphenicol etc. plus multidrug resistance; for the full list, please refer to Additional file 4.

The analysis of potential antibiotic resistance shows that the three *K. oxytoca* strains have similar antibiotic resistance profiles with the exceptions of resistance to gentamincin_b, kasugamycin, trimethoprim, and dibekacin. Dibekacin resistance was found only in *K. oxytoca* E718, while resistance to kasugamycin was not found in *K. oxytoca* KCTC 1686. This comparative study provides detailed antibiotic resistance profiles for *K. oxytoca* strains that will be useful for future gene/gene cluster knockout experiments.

*K. oxytoca* was reported as a bacterial species resistant to several antibiotics (Högenauer 2006). Potential antibiotic resistant genes were identified in all three *K. oxytoca* complete genomes. The number of potential antibiotic genes and the antibiotic categories are similar in all three *K. oxytoca* strains. For *K. oxytoca* HKOPL1, 12 potential antibiotic resistant genes may come from plasmid and one potential antibiotic gene may come from prophage (data not shown); this information is important for studying antibiotic resistant genes existing in the gut microbiota of giant pandas. Future experiments are required to confirm all potential antibiotic resistance in *K. oxytoca* HKOPL1.

### Potential mobile elements in *K. oxytoca*HKOPL1

About 28% (1,615 out of 5,772) of the predicted genes have been identified in the ACLAME database. For *K. oxytoca* HKOPL1, 20 genes potentially come from viruses, 1,398 genes potentially come from plasmids and 197 genes potentially come from prophages. The total number of horizontal gene transferring (HGT) elements in *K. oxytoca* HKOPL1 is less than the other two closely related strains (Table 3). Twenty prophage associated genes were further curated to show their functions as well as their sources. Results indicate that all 20 prophage associated genes are from different phages infecting a wide range of bacteria genus including *Aeromonas, Enterobacteria, Xanthomonas, Staphylococcus, Geobacillus, Enterococcus, Prochlorococcus, Vibrio, Mannheimia, Klebsiella* and *Salmonella* (see Additional file 5). Some of the phage genes, such as contig00001_orf00136/contig00001_orf00138 (NrdD anaerobic NTP reductase large/small subunit) and contig00001_orf05069/contig00001_orf05192/contig00001_orf05193/contig00001_orf05194/contig00001_orf05202 (Gp22, Gp18, Gp18, Gp20 and Gp62), are clustered together. Such evidence suggests that these clustered genes were probably obtained from ancient phage integration events even though these regions lack complete phage structure (see Additional file 5).

**Table 3:** **Comparative analysis of number of potential HGT genes between** ***K. oxytoca*** **HKOPL1 and 2 closely related** ***K. oxytoca*** **strains**

HGT vector type	***K. oxytoca*** HKOPL1	***K. oxytoca*** KCTC 1686	***K. oxytoca*** E718
Prophage associated	20	43	58
Plasmid	1398	1397	1425
Prophage	197	219	252
Total	1615	1659	1735

HGT events were very common in bacteria species [14] and prophages, viruses and plasmids were supposed to be major vectors in HGT events [14]. For *K. oxytoca* HKOPL1*,* 28% of its genes were identified as potential HGT genes. The number of potential HGT genes in the other two *K. oxytoca* strains was similar. Most of the potential HGT genes of *K. oxytoca* HKOPL1 might have come from plasmids (~80%).

A region ranging from 4,612,823bp to 4,641,509bp was identified as an incomplete prophage region (see Additional file 2). The length of this region was 28.6kb with 39 CDS and it was similar to Enterobacterial phage mEp390. The CDS in this region was classified into different categories and illustrated in Additional file 6.

Horizontal gene transfer analysis suggests that HGT events are common in all *K. oxytoca* strains and various plasmids can be potential players in such events. In addition, 20 prophage associated genes were identified in *K. oxytoca* HKOPL1 from various phages infecting a wide range of different bacteria species. Some of them were clustered together, suggesting that they were obtained from ancient phage integration events. An incomplete prophage integrated region (~28.6 kb) was also identified in *K. oxytoca* HKOPL1 which might have been obtained from a previous prophage integration event.

### Pathway analysis of *K. oxytoca*HKOPL1

Functional pathways of *K. oxytoca* HKOPL1 were annotated using KEGG Automatic Annotation Server. Among all these pathways, the starch and sucrose metabolism pathway (ko00500) and the butanoate metabolism pathway (ko00650) were present in the annotation result.

For the starch and sucrose metabolism pathway (ko00500), 32 genes were identified and two of them are related to cellulose degradation. They are Endoglucanase (K01179, EC:3.2.1.4) and Beta-glucosidase (K05349, EC:3.2.1.21). The former enzyme is able to transform cellulose to cellobiose, while the latter enzyme is able to transform cellobiose to β-D-Glucose. Further comparative analysis also shows that endoglucanase and beta-glucosidase are core genes existing in all 10 *Klebsiella* strains. This evidence reveals that *Klebsiella* may be able to degrade/utilize cellulose as an energy source and might have the ability to survive cellulose-rich environments such as inside the gut of giant pandas.

For the butanoate metabolism pathway (ko00650), 33 genes were identified and some of them were related to 2,3-Butanediol production. 2,3-Butanediol is widely used in plastics, solvents and antifreeze preparation. The 2,3-Butanediol production pathway is as follows: 1) Pyruvate dehydrogenase E1 component subunit alpha/beta (K00161 and K00162, EC:1.2.4.1) and Pyruvate dehydrogenase E1 component (K00163, EC:1.2.4.1) convert pyruvate to 2-(α-Hydroxyethyl)-ThPP, 2) Acetolactate synthase I/II/III large/small subunit (K01652 and K01653, EC:2.2.1.6) and acetolactate synthase II small subunit (K11258, EC:2.2.1.6) convert 2-(α-Hydroxyethyl)-ThPP to 2-Acetolactate, 3) Acetolactate decarboxylase (K01575, EC:4.1.1.5) converts 2-Acetolactate to (R)-2-Acetoin, and 4) (R,R)-butanediol dehydrogenase / diacetyl reductase (K03366, EC:1.1.1.4) convert (R)-2-Acetoin to 2,3-Butanediol. Further comparative analysis shows that all genes in the 2,3-Butanediol production pathway are core genes existing in all 10 *Klebsiella* strains. This evidence reveals that *Klebsiella* may be a bacterial genus that has industrial potential for 2,3-Butanediol production (e.g. a 2,3-Butanediol producing strain *K. pneumonia* KCTC 2242 [7]).

Pathway analysis identified a cellulose degradation pathway in *K. oxytoca* HKOPL1*.* Further comparative study shows that this cellulose degradation pathway is present in all 10 *Klebsiella* strains. This evidence reveals that *Klebsiella* may have the ability to degrade/utilize cellulose as an energy source and survive cellulose-rich environments, and it also provides insight into speculation that the gastrointestinal bacteria of giant pandas help to transform cellulose to β-D-Glucose, which is absorbed by the gastrointestinal cells as an energy source. Since giant pandas cannot degrade bamboo cellulose using its own digestive enzymes [2], *K. oxytoca* HKOPL1 may play a key role in supplying energy to giant pandas. This is the first report with evidence of cellulose metabolism in a complete bacteria genome isolated from giant panda feces. In addition, a 2,3-Butanediol production pathway was identified in *K. oxytoca* HKOPL1 and found in all 10 *Klebsiella* strains as well. This discovery is of particular industrial interest because it means the *Klebsiella* genus could potentially produce 2,3-Butanediol in a biologically sustainable way.

### Cellulose degradation ability on CMC Agar plate

To provide functional evidence of *K. oxytoca* HKOPL1’s ability to degrade cellulose, carboxymethyl cellulose (CMC) was used as a sole carbon source. After 72 hours of anaerobic incubation at 37°C, the appearance of a clear zone around the colony was observed (see Additional file 7). This result infers the presence of cellulolytic activity of isolated *K. oxytoca* HKOPL1 from the gut of giant pandas.

## Conclusions

*K. oxytoca* HKOPL1, isolated from giant panda feces, has a circular genome of 5.9 Mb. Annotations against various databases, phylogenomic analysis and comparative genomic studies were done on this complete genome. *K. oxytoca* HKOPL1 was found to have a closer relationship with *K. oxytoca* strains 1686 and E718 than with the *K. pneumoniae* strains. The percentages of all COG classes are similar within the three *K. oxytoca* strains. A couple of virulence genes along with PAI-like regions were identified in *K. oxytoca* HKOPL1*.* A detailed antibiotic resistance profile for *K. oxytoca* strains was illustrated based on a comparative study. Our study also suggests that HGT events are common in all *K. oxytoca* strains, and plasmids are potentially important players in such events. In addition, both ancient and recent potential phage/prophage integration events were identified in *K. oxytoca* HKOPL1*.* Pathway analysis shows that there are two important pathways existing in *K. oxytoca* HKOPL1 that contribute to cellulose degradation and 2,3-Butanediol production. Cellulose degradation activity of *K. oxytoca* HKOPL1 was confirmed by functional screening on solid cellulose media. Further comparative analysis shows that these two important pathways can be identified in all 10 *Klebsiella* strains.

In summary, this present work has done both comprehensive genomic study and comparative genomic study based on the complete genome of *K. oxytoca* HKOPL1*.* As a result, *K. oxytoca* HKOPL1, as well as other strains in the *Klebsiella* genus, may have a lot of potential in the biological industry.

## Methods

### Bacterial strain

*K. oxytoca* HKOPL1 was isolated from a healthy adult giant panda living in the zoological institution of Hong Kong. All samples were obtained with permission and under memorandum through the Ocean Park Corporation, Hong Kong. First, fecal swabs were taken from inside fresh fecal masses using sterile plain swabs (Copan®#155C) and then streaked on different primary culture media. Afterwards, the agar plates were incubated aerobically at 37°C for 18–24 hours. Initial colonies were separated from the rest according to the colonial morphology on the primary plates, and then Gram staining was performed on these colonies. Subcultures of each colony from each agar medium were performed in the appropriate media (e.g. brain heart infusion agar plate - Oxoid® #CM1136) to get the pure culture. This was followed by initial rapid identification procedures (catalase, oxidase, indole tests etc.) and biochemical identification using Mini API® and API® systems (bioMerieux ®sa France).

### Total DNA extraction of *K. oxytoca*HKOPL1

An isolated single bacteria colony was grown in LB medium overnight to reach the exponential growth phase. Bacteria cells were then harvested by centrifugation before performing the bacteria genomic DNA extraction according to JGI’s “Bacterial genomic DNA isolation using CTAB” protocol [15].

### 454 Pyrosequencing of the *K. oxytoca*HKOPL1 genome and sequence assembly

To confirm the purity of genomic DNA, the 16S rDNA specific region of *K. oxytoca* HKOPL1 was amplified and cloned. Then, 20 positive clones were submitted for Sanger sequencing. BLASTN analysis revealed that the *K. oxytoca* HKOPL1 16S rDNA sequences highly correlate with the current *Klebsiella* genus database. Evaluation of the quality of genomic DNA was done by using the Quant-iT™ PicoGreen dsDNA kit (Invitrogen).

A whole genome shotgun library of *K. oxytoca* HKOPL1 was generated with 0.5 μg of the genomic DNA; the shotgun sequencing was done with 454 GS Junior General Library Preparation Kit (Roche). In addition, an 8kb insert paired-end library was generated with 15 μg of the genomic DNA; the paired-end sequencing was done with 454 GS Junior Paired-end Library Preparation Kit (Roche). Paired-end reads were used to orientate the contigs into scaffolds.

The DNA libraries were amplified by emPCR and sequenced with FLX Titanium Sequencing Chemistry (Roche). Two shotgun runs and two paired-end runs were performed for each library. After sequencing, the raw data was assembled by Newbler 2.7 (Roche) with default parameters. Primer pairs were designed to amplify the gaps between contigs, and the resulting PCR products were directly sequenced by Sanger sequencer ABI 3130.

### Genome annotation of *K. oxytoca*HKOPL1

To predict the genes in the complete genome of *K. oxytoca* HKOPL1, Glimmer 3.02 [16], a prokaryotic gene prediction software, was used. First, all predicted CDS (Coding DNA Sequences) were translated into protein sequences by in-house Perl scripts. These protein sequences were then aligned against the NCBI non-redundant database (January 2013) with BLASTP [17]. Filtering of protein sequences with >90% alignment length and >40% identity was applied, and the description of the best hit was assigned to each corresponding predicted gene. Intergenic regions were annotated by RepeatMasker [18] with default parameters.

### Phylogenomic analysis of *K. oxytoca*HKOPL1

In order to identify the evolutionary position of *K. oxytoca* HKOPL1, phylogenomic analysis was applied to this strain together with other publicly available *Klebsiella* strains. Nine complete genomes of *Klebsiella* strains were downloaded from NCBI GenBank (see Additional file 8 for accession numbers). The orthologous genes were identified with BLAT (BLAST-like alignment tool, [19]) under default parameters, by aligning the predicted genes of *K. oxytoca* HKOPL1 against all annotated genes of the nine *Klebsiella* strains.

The single copy genes with >90% of alignment length of the total *Klebsiella* strains were considered core genes. All the core genes were then aligned by MUSCLE (multiple sequence comparison by log-expectation) under default parameters [20] and concatenated together. Finally, the concatenated aligned genes were submitted to MrBayes [21] with the GTR + G + I substitution model for BMCMC (Bayesian Markov Chain Monte Carlo) phylogenomic tree construction. The chain length was set to 10,000,000 (1 sample per 1,000 generations). The first 2,000 samples were discarded as burn in after scrutinizing the trace files of two independent runs with Tracer v1.4 [22].

For comparative studies, the orthologous genes that were found to be shared between *K. oxytoca* HKOPL1 and other *Klebsiella* strains were plotted alongside all the predicted genes in *K. oxytoca* HKOPL1 in order to compare pathogenicity island-like regions and prophage-like regions in *K. oxytoca* HKOPL1. For comparative study within *K. oxytoca* species only, a Venn diagram was constructed based on the common genes found in the three *K. oxytoca* strains (*K. oxytoca* HKOPL1*, K. oxytoca* KCTC 1686 *and K. oxytoca* E718). Circular figures were generated with CIRCOS software [23].

### COG analysis of *K. oxytoca*HKOPL1

The COG (Clusters of Orthologous Groups of proteins) database [24] is a collection of functionally orthologous proteins from both prokaryotic and eukaryotic genome sequences. This database is often applied to functional gene classification of complete bacterial genome sequences.

In this study, the predicted ORF (Open Reading Frame) sequences of *K. oxytoca* HKOPL1 were translated into protein sequences by in-house Perl scripts. BLASTP [17] was subsequently applied to align these translated protein sequences against the COG database. Protein sequences with >90% alignment length and >20% identity were filtered, with the description of the best hit assigned to the corresponding predicted gene. Then, all predicted genes were classified into COG classes. The *K. oxytoca* HKOPL1 COG annotation was also compared to the other two *K. oxytoca* strains.

### Virulence gene analysis of *K. oxytoca*HKOPL1

The Virulence Factor Database (VFDB) [25] is a collection of virulence factors (VFs) of various medically significant bacterial pathogens. *In silico* identification of virulence factors is helpful prior to any form of virulence-based animal experiment or gene knock-out experiment.

As in COG analysis, the predicted ORF sequences of *K. oxytoca* HKOPL1 were translated into protein sequences by in-house Perl scripts. BLASTP [17] was subsequently applied to align these protein sequences against the VFDB database. Protein sequences with >90% alignment length and >40% identity were filtered, and the description of the best hit was assigned to the corresponding predicted gene. *K. oxytoca* HKOPL1 virulence factors were compared to other publicly available *Klebsiella* strains.

Pathogenicity islands (PAIs) are mobile microbial genetic elements essential for the process of disease development; they are important research targets of microbial pathogenesis. They have been observed in evolutionary distant bacteria and may have been horizontally transferred among microbes. The pathogenicity island database (PAIDB) [26] is a comprehensive database of all reported pathogenicity islands (PAIs) and predicted/potential PAI regions. *In silico* identification of PAIs is useful prior to subsequent confirmation by gene knock-out experiments. In this study, all predicted genes of *K. oxytoca* HKOPL1 were preprocessed by in-house Perl scripts before submission to PAIDB for the identification of pathogenicity island-like regions.

### Drug resistant gene analysis of *K. oxytoca*HKOPL1

Bacterial antibiotic resistance is often caused by bacteria acquiring various drug resistant genes. The Antibiotic Resistance Genes Database (ARDB) [27] is a summary of most antibiotic resistance genes from publicly available information. Each gene (including its resistance type) is annotated with its resistance profile and mechanism of action.

To analyze the drug resistant genes of *K. oxytoca* HKOPL1, in-house Perl scripts were used to translate predicted ORF sequences into protein sequences. These protein sequences were then submitted to BLASTP [17] to be aligned against the ARDB database. Protein sequences with >90% alignment length and >40% identity were filtered, and the description of the best hit is assigned to the corresponding predicted gene. Lastly, in-house Perl scripts were used again to summarize all annotated genes for their corresponding antibiotics. *K. oxytoca* HKOPL1’s drug resistant genes were compared to the other two *K. oxytoca* strains.

### Potential mobile elements and prophage-like regions identification analysis of *K. oxytoca*HKOPL1

The major source of prokaryotic horizontal gene transferring (HGT) elements includes genes from phage genomes, plasmids, viruses and transposons. Classification of these genes can be found in the ACLAME (A CLAssification of Mobile genetic Elements) database [28], which helps scientists to discover potential HGT genes from various sources. Psi-BLAST and HMM (Hidden Markov Model) methods were applied in a similarity search against the public database to classify HGT genes into different sources.

As before, analysis starts with translating predicted ORF sequences of *K. oxytoca* HKOPL1 into protein sequences by in-house Perl scripts. BLASTP [17] was then applied to align all these protein sequences against the ACLAME database. Protein sequences with >90% alignment length and >40% identity were filtered, and the description of the best hit is assigned to each corresponding predicted gene before matching all annotated genes to their corresponding horizontal transferring vectors. *K. oxytoca* HKOPL1’s potential horizontal transferring genes were again compared with the other two *K. oxytoca* strains.

In relation to prophage-like regions, phages are viruses which infect bacteria and they can be classified into two categories: lytic and temperate. Integrated phages are technically termed as prophages. Temperate phages are able to integrate into the plasmid or chromosome of their bacterial host. The PHAge Search Tool (PHAST) is a web server for identification of prophage existence (both complete and incomplete) [29]. The complete genome sequence of *K. oxytoca* HKOPL1 was submitted to PHAST for prophage-like region identification. Afterwards, the features of the prophage-like region were summarized.

### Pathway analysis of *K. oxytoca*HKOPL1

Kyoto Encyclopedia of Genes and Genomes (KEGG) is a high level pathway analysis database which collects metabolic pathway information and integrate genomic, chemical and systemic functional information [30]. KEGG gene catalogs from genomes are associated with higher systemic cell functions. To analyze the metabolic pathway of *K. oxytoca* HKOPL1, the predicted ORF sequences were first translated into protein sequences by in-house Perl scripts. Then, these protein sequences were submitted to the KEGG database for automatic pathway annotation (http://www.genome.jp/kaas-bin/kaas_main). Afterwards, all annotated pathways were manually downloaded and curated by in-house Perl scripts.

### Detection of cellulose degradation ability on agar plate

Five microliters of pure culture grown overnight (OD600 ~ 1) was spotted on CMC (Carboxymethylcellulose) agar plates, which were prepared according to the procedure reported by Kasana [31]. Plates were incubated anaerobically at 37°C for 72 hours, followed by flooding with Gram’s Iodine solution for 3 minutes. The appearance of a clear zone around the colony is recognized for the bacteria’s ability to degrade CMC.

### Availability of supporting data

The data set supporting the results of this article is available in the NCBI BioProject repository (accession: CP004887, BioProject ID: PRJNA194061), unique persistent identifier and hyperlink to dataset in http://www.ncbi.nlm.nih.gov/bioproject/194061.

## Electronic supplementary material

Additional file 1: **Comparative study on common genes of** ***Klebsiella*** **strains shared with** ***K. oxytoca*** **HKOPL1.** (XLSX 11 KB)

Additional file 2: **Comparative CDS analysis for 10** ***Klebsiella*** **strains.** From inside to outside, GC content %, predicted CDS in *K. oxytoca* HKOPL1 (pink), CDS of *K. oxytoca* KCTC 1686 shared with K*. oxytoca* HKOPL1 (pink), CDS of *K. oxytoca* E718 shared with *K. oxytoca* HKOPL1 (pink), CDS of *K. pneumoniae subsp. pneumoniae* MGH 78578 shared with *K. oxytoca* HKOPL1 (blue), CDS of *K. pneumoniae* 342 shared with *K. oxytoca* HKOPL1 (blue), CDS of *K. pneumoniae subsp. pneumoniae* NTUH-K2044 shared with *K. oxytoca* HKOPL1 (blue), CDS of *K. pneumoniae subsp. pneumoniae* HS11286 shared with *K. oxytoca* HKOPL1 (blue), CDS of *K. pneumoniae* KCTC 2242 shared with *K. oxytoca* HKOPL1 (blue), CDS of *K. pneumoniae subsp. pneumoniae* 1084 shared with *K. oxytoca* HKOPL1 (blue), CDS of *K. pneumoniae* 342 shared with *K. oxytoca* HKOPL1 (blue), CDS of *K. variicola* At-22 shared with *K. oxytoca* HKOPL1 (orange), and pathogenicity island-like region (red)/potential prophage integration region (green) identified in *K. oxytoca* HKOPL1 are plotted according to their scales. (PDF 4 MB)

Additional file 3: **Features of Pathogenicity island-like regions in** ***K. oxytoca*** **HKOPL1.** (XLSX 18 KB)

Additional file 4: **Number of potential drug resistant genes in 3** ***K. oxytoca*** **strains.** (PDF 39 KB)

Additional file 5: **Prophage associated genes identified in** ***K. oxytoca*** **HKOPL1 and their sources.** (XLSX 11 KB)

Additional file 6: **CDS in Prophage integration region of** ***K. oxytoca*** **HKOPL1.** (PDF 33 KB)

Additional file 7: **Detection of cellulose activity for** ***K. oxytoca*** **HKOPL1.** Carboxymethylcellulose (CMC) agar plates were inoculated with 5 μL of pure culture (OD600 ~ 1) and after 72 hours of anaerobic incubation, flooded with Gram’s Iodine. (PDF 4 MB)

Additional file 8: ***Klebsiella*** **strains information.** (XLSX 10 KB)

## Abbreviations

*K.*: *Klebsiella*
CDS: Coding DNA sequence
ORF: Open reading frame
PAI: Pathogenicity Island
COG: Clusters of orthologous groups of proteins
VFDB: Virulence factor database
PAIDB: Pathogenicity Island database
ARDB: Antibiotic resistance genes database
ACLAME: A CLAssification of mobile genetic elements
PHAST: PHAge search tool
KEGG: Kyoto encyclopedia of genes and genomes
HGT: Horizontal gene transfer
BMCMC: Bayesian Markov Chain Monte Carlo.
