# Supplementary figures and images for: Complete genome sequence and comparative genome analysis of Klebsiella oxytoca HKOPL1 isolated from giant panda feces
Source: BMC Res Notes. 2014 Nov 23;7:827. doi: 10.1186/1756-0500-7-827 (PMC4289185; doi:10.1186/1756-0500-7-827)

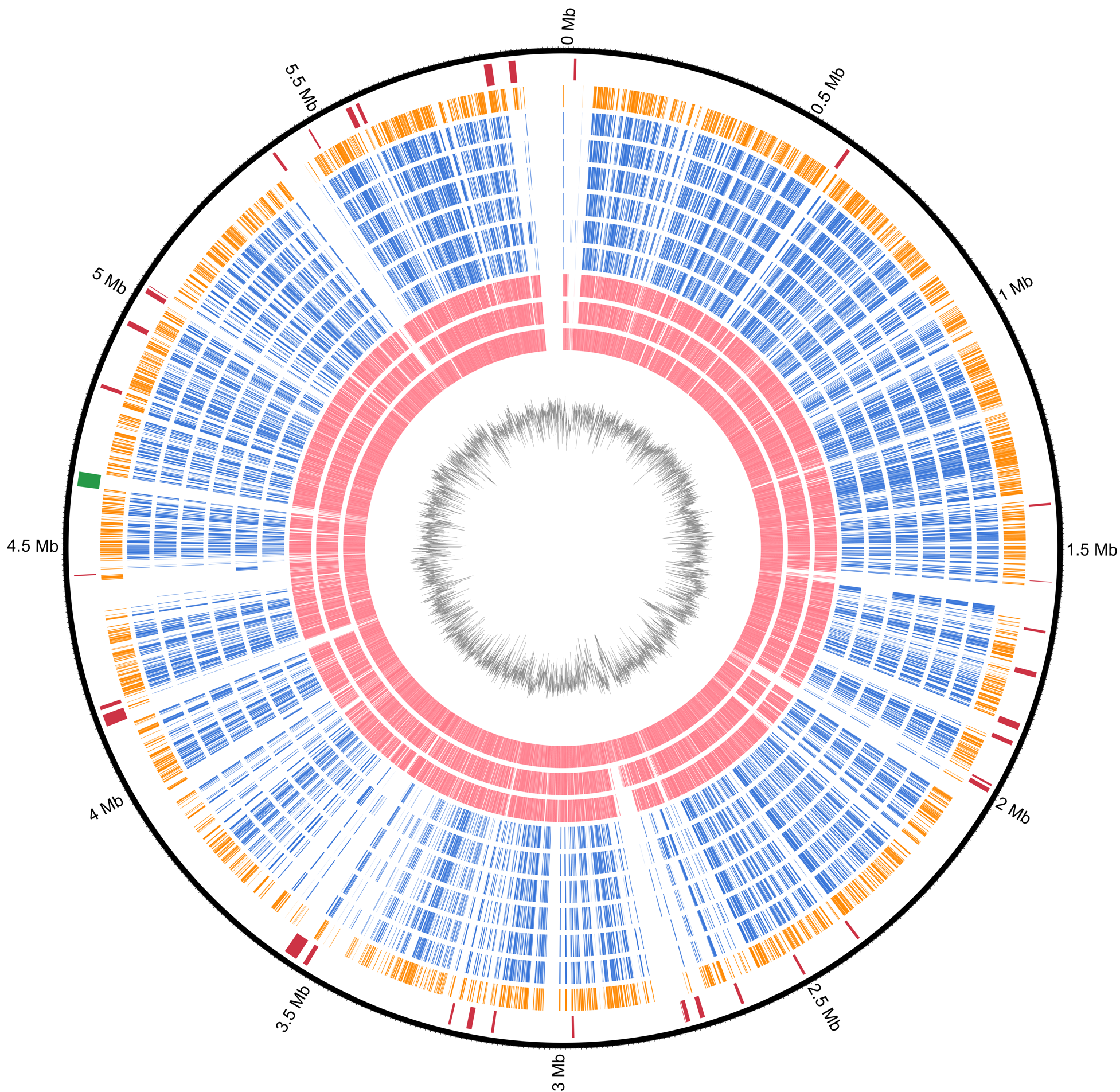

Supplement: Supplementary file 2 — Additional file 2: Comparative CDS analysis for 10 Klebsiella strains. From inside to outside, GC content %, predicted CDS in K. oxytoca HKOPL1 (pink), CDS of K. oxytoca KCTC 1686 shared with K. oxytoca HKOPL1 (pink), CDS of K. oxytoca E718 shared with K. oxytoca HKOPL1 (pink), CDS of K. pneumoniae subsp. pneumoniae MGH 78578 shared with K. oxytoca HKOPL1 (blue), CDS of K. pneumoniae 342 shared with K. oxytoca HKOPL1 (blue), CDS of K. pneumoniae subsp. pneumoniae NTUH-K2044 shared with K. oxytoca HKOPL1 (blue), CDS of K. pneumoniae subsp. pneumoniae HS11286 shared with K. oxytoca HKOPL1 (blue), CDS of K. pneumoniae KCTC 2242 shared with K. oxytoca HKOPL1 (blue), CDS of K. pneumoniae subsp. pneumoniae 1084 shared with K. oxytoca HKOPL1 (blue), CDS of K. pneumoniae 342 shared with K. oxytoca HKOPL1 (blue), CDS of K. variicola At-22 shared with K. oxytoca HKOPL1 (orange), and pathogenicity island-like region (red)/potential prophage integration region (green) identified in K. oxytoca HKOPL1 are plotted according to their scales. (PDF 4 MB) [file 13104_2014_3426_MOESM2_ESM.pdf]

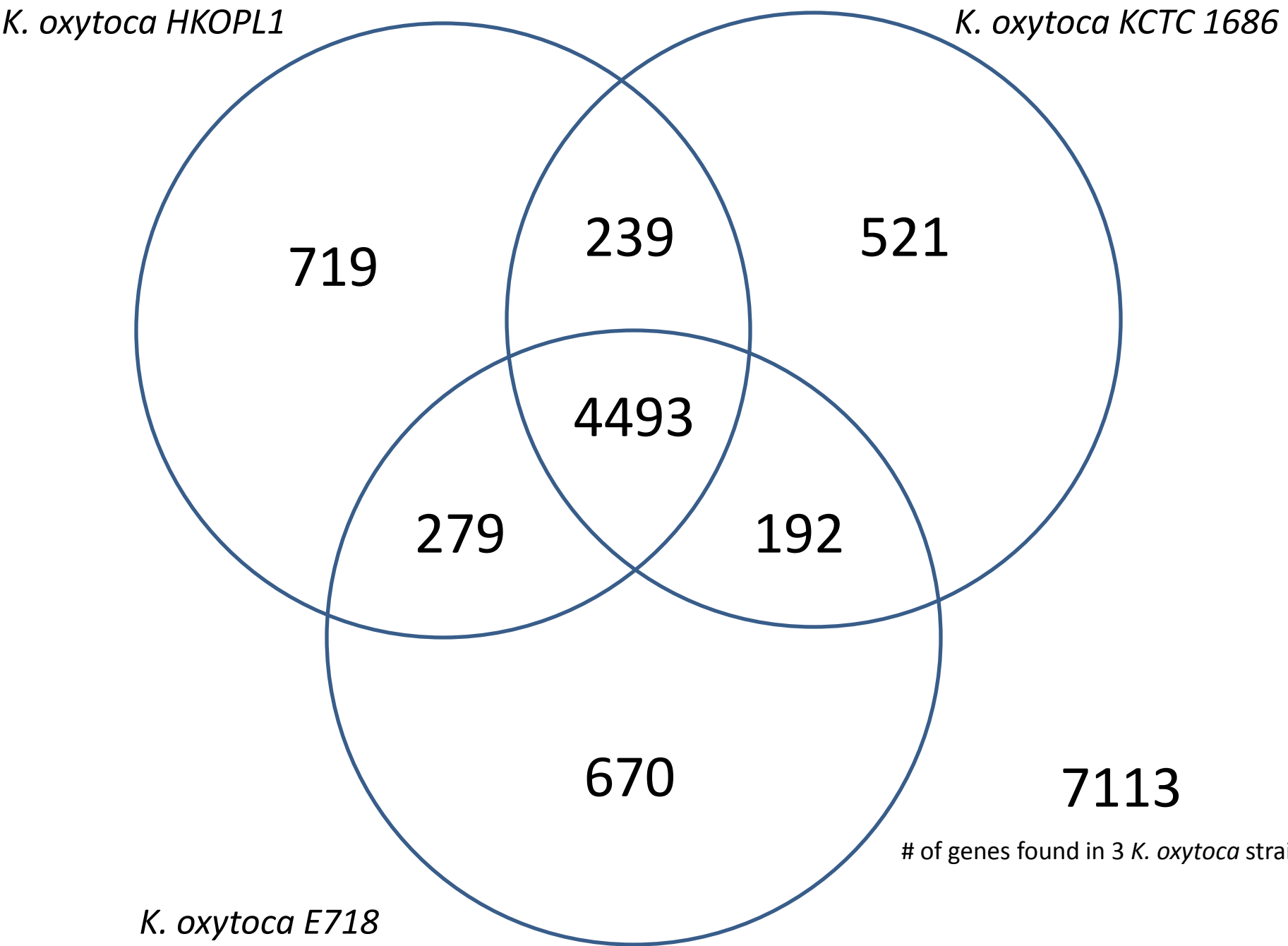

7113

# of genes found in 3 *K. oxytoca* strains

Supplement: Supplementary file 4 — Additional file 4: Number of potential drug resistant genes in 3 K. oxytoca strains. (PDF 39 KB) [file 13104_2014_3426_MOESM4_ESM.pdf]

# Prophage region

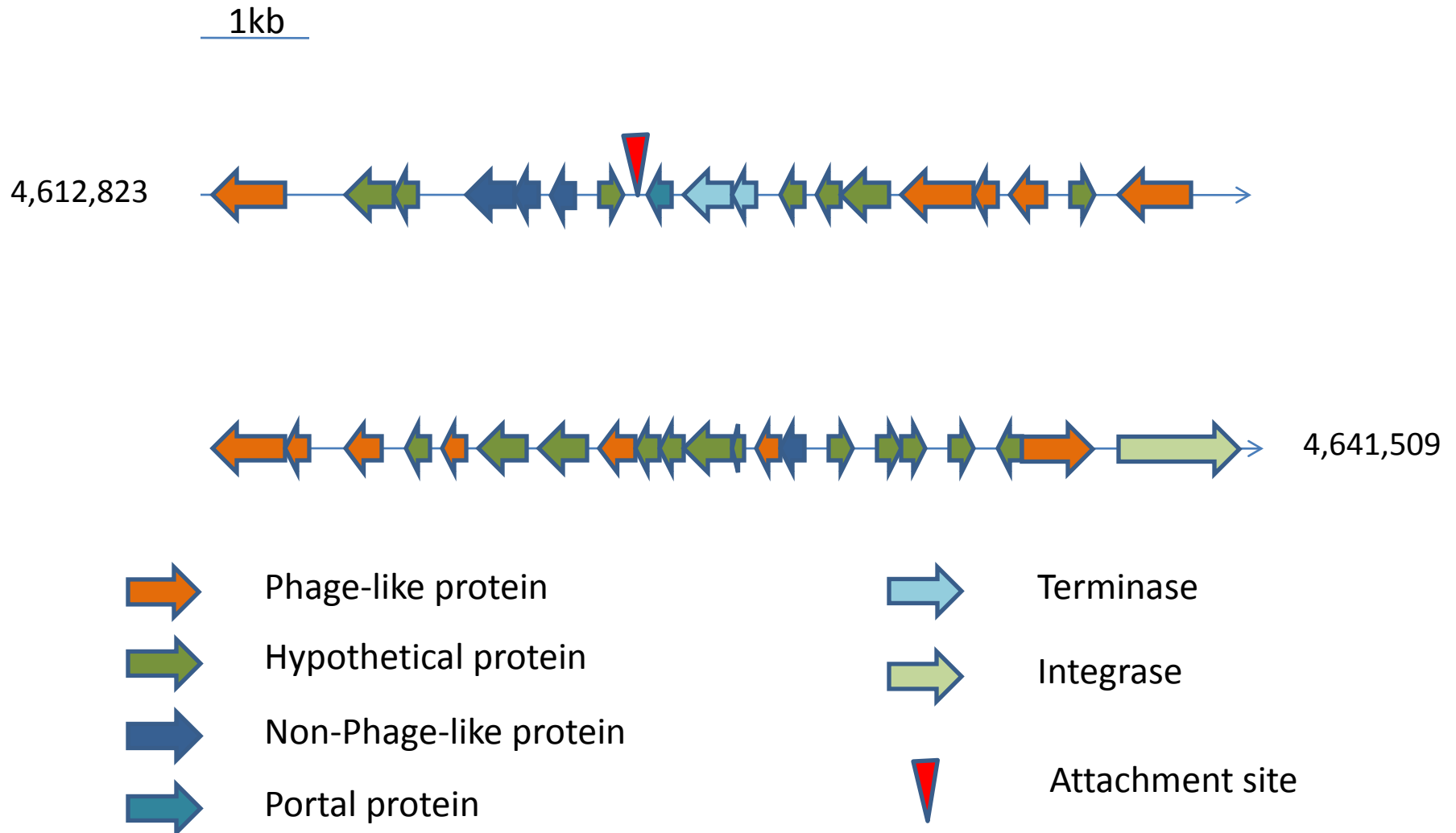

Supplement: Supplementary file 6 — Additional file 6: CDS in Prophage integration region of K. oxytoca HKOPL1. (PDF 33 KB) [file 13104_2014_3426_MOESM6_ESM.pdf]
