# Supplementary material for: Complete genome sequence and comparative genome analysis of Klebsiella oxytoca HKOPL1 isolated from giant panda feces
Source: BMC Res Notes. 2014 Nov 23;7:827. doi: 10.1186/1756-0500-7-827 (PMC4289185; doi:10.1186/1756-0500-7-827)

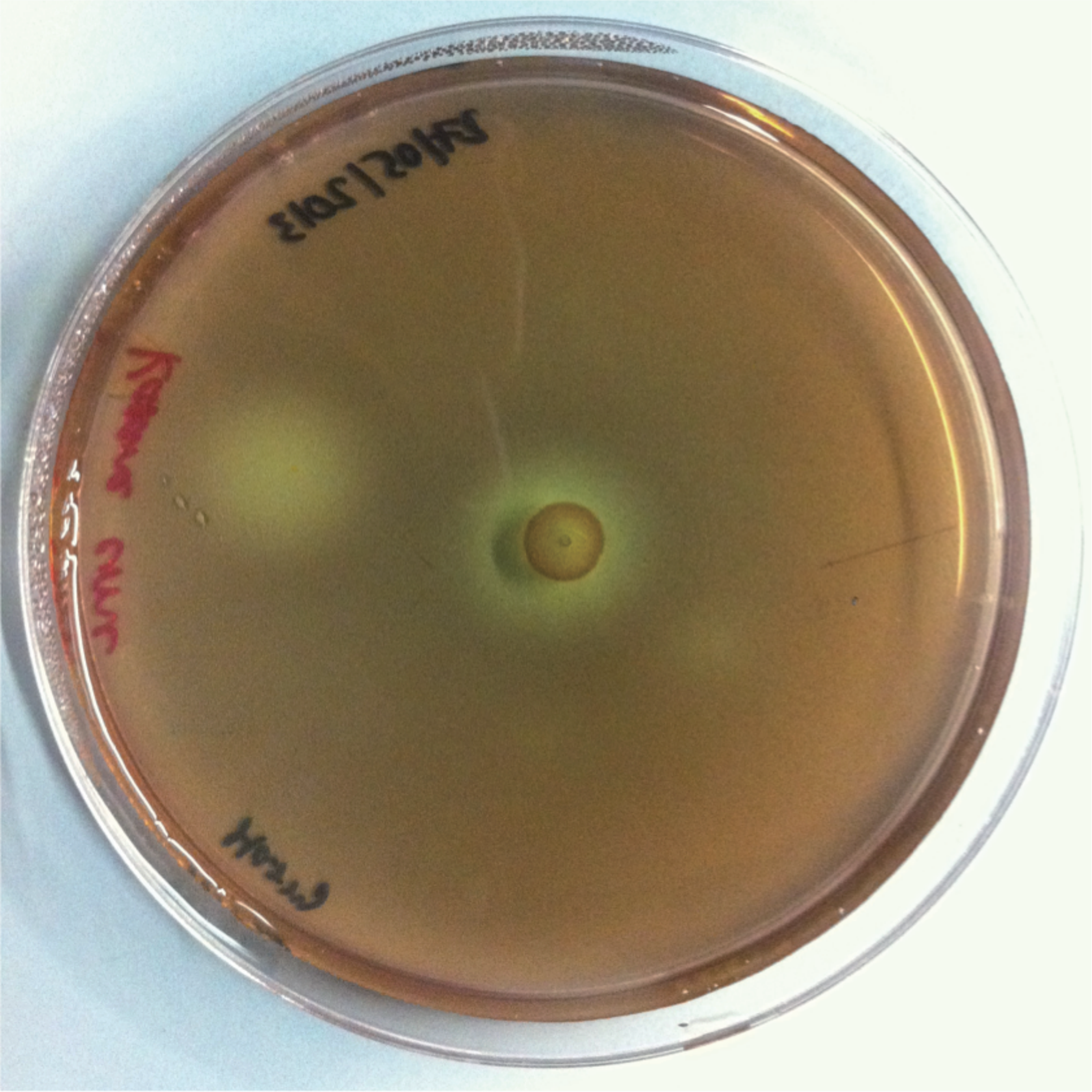

Supplement: Supplementary file 7 — Additional file 7: Detection of cellulose activity for K. oxytoca HKOPL1. Carboxymethylcellulose (CMC) agar plates were inoculated with 5 μL of pure culture (OD600 ~ 1) and after 72 hours of anaerobic incubation, flooded with Gram’s Iodine. (PDF 4 MB) [file 13104_2014_3426_MOESM7_ESM.pdf]
